# Post-operated case of mucormycosis: a rare image

**DOI:** 10.11604/pamj.2022.42.247.35905

**Published:** 2022-08-02

**Authors:** Himanshi Krushna Sathawane, Manjusha Mahakalkar

**Affiliations:** 1 Department of Obstetrics and Gynecological Nursing, Smt. Radhikabai Meghe Memorial College of Nursing, Datta Meghe Institute of Medical Sciences, Sawangi, Wardha, Maharashtra, India

**Keywords:** Mucormycosis, black fungus, lesion, endoscopic debridement

## Image in medicine

Mucormycosis also known as black fungus, it is relatively rare, but also very serious. It commonly infects the nose, sinuses, eye, and brain resulting in a runny nose, one-sided facial swelling and pain, headache, fever, blurred vision, bulging or displacement of the eye (proptosis), and tissue death. Other forms of disease may infect the lungs, stomach and intestines, and skin. In India, prevalence of mucormycosis is approximately 0.14 per 1000 population. A 58-year-old male came with the complaints of severe headache since a week, nasal congestion, black lesion on nasal bridge and upper inside of mouth that progressively became severe within 2 weeks. Physician inspected the patient and suggested for computed tomography (CT) and magnetic resonance imaging (MRI) scans. After all investigations patient was diagnosed as a case of mucormycosis and had undergone endoscopic debridement.

**Figure 1:**
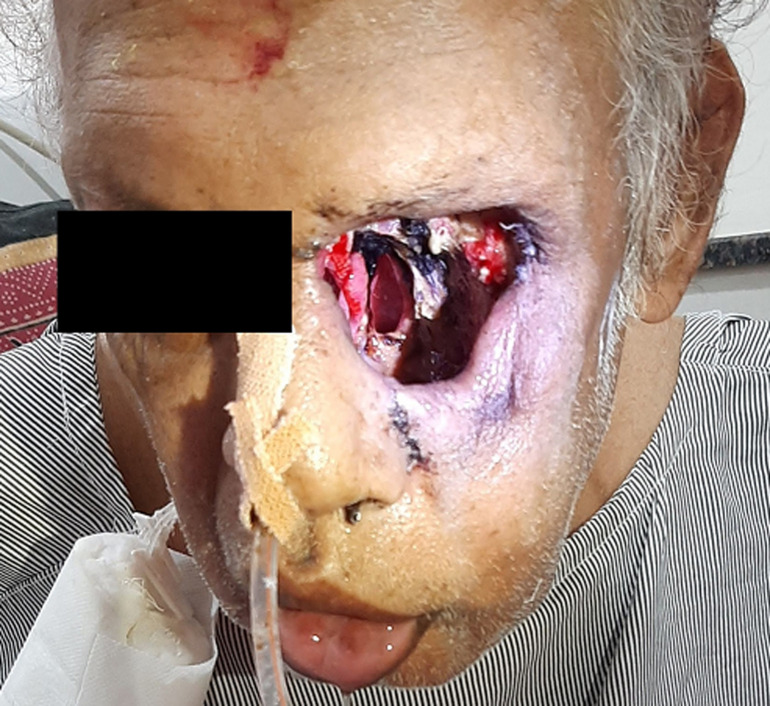
clinical image showing post-operated mucormycosis extended to left eye

